# The use of Hall technique preformed metal crowns by specialist paediatric dentists in the UK

**DOI:** 10.1038/sj.bdj.2018.4

**Published:** 2018-01-12

**Authors:** A Roberts, A McKay, S Albadri

**Affiliations:** 1 Clinical Lecturer in Paediatric Dentistry, School of Dentistry University of Liverpool Pembroke Place, Liverpool, Merseyside, L3 5PS, United Kingdom; 2 Specialist in Paediatric Dentistry, Rotherham Community Dental Service, Rotherham Community Health Centre, Greasbrough Road, Rotherham, S60 1RY; 3 Reader/Honourary Consultant in Paediatric Dentistry, Deputy Head of School of Dentistry, Teaching and Clinical Lead for Paediatric Dentistry, School of Dentistry University of Liverpool, Pembroke Place, Liverpool, Merseyside, L3 5PS, United Kingdom.

## Abstract

**Background** Hall technique preformed metal crowns (HTPMCs) have been increasing in use recently, but little is currently known about their use by specialists.

**Aim** To investigate the views and usage of HTPMCs by UK specialist paediatric dentists.

**Design** This was a prospective questionnaire-based study, distributed online to all specialists on the British Society of Paediatric Dentistry email list between July and September 2014.

**Results** Ninety-four questionnaires were completed. The majority of respondents, 65% (61) worked in teaching hospitals, followed by community dental services, 37% (35). Ninety-six percent (89) reported that they used HTPMCs in their practice. Fifty-eight percent (54) used HTPMCs as a treatment option for restoring symptomless carious primary molars, and 15% (14) only when unable to provide conventional restoration. Twenty-three percent (21) used HTPMCs as the treatment of choice. Only 4% (4) of respondents never used them. Sixty percent (53) had been using HTPMCs for over five years. Seventy-six percent (68) would consider placing HTPMCs under inhalation sedation, and 26% (23) under general anaesthesia. Over 90% (85) believed that HTPMCs are suitable for undergraduate teaching, general practice, postgraduate training and specialist practice.

**Conclusion** HTPMCs are widely used among specialist paediatric dentists in the UK.

## Introduction

This study aimed to understand the current use of HTPMCs by specialists in paediatric dentistry, in order to understand in which clinical situations they are using them. It is hoped that this knowledge will aid those less familiar and less confident with the technique, to make decisions around when it is appropriate to use HTPMCs. Although clear guidelines are given within the Hall technique manual,^1^ if dentists are aware of the clinical situations in which specialists are using HTPMCs, it may aid them to make decisions about their use in particular situations.

The Hall technique has been described as being the biggest breakthrough in paediatric dentistry research in the last ten years, one that will revolutionise paediatric dentistry.^2^ A Hall technique crown is one in which there has been no preparation to the tooth, with caries sealed in, and is used on primary molars as an alternative to conventional restoration. This technique was developed by a general dental practitioner (GDP) in Scotland, Dr Hall, to allow use of preformed metal crowns without any preparation to the tooth. A detailed description of the technique is described in the manual.^1^

Before the Hall technique, crowns fitted on primary molars required local anaesthetic and crown preparation; something that is not necessarily practical for very young children. HTPMCs do not require local anaesthetic or any preparation and can be used on children as young as three years old.

The rationale behind the success of HTPMCs is based on sealing in caries rather than removing it. This works by denying biofilm microbes their source of nutrition, dietary carbohydrate, and removing this access prevents progression of caries.^3^,^4^ The dental pulp is then able to lay down reparative dentine. A recent systematic review demonstrated the advantage of avoiding caries removal in terms of preventing pulpal exposure.^5^

A systematic review in 2000 showed, in all ten studies reviewed, that preformed metal crowns (PMCs) outperformed amalgam restorations for class two cavities.^6^ All further studies to date suggest that PMCs outperform conventional restorations when used to treat carious lesions involving more than one surface, and they are therefore recommended in the UK National Clinical Guidelines in Paediatric Dentistry^7^ as the treatment of choice for lesions of two or more surfaces, and extensive one surface lesions.

Findings of the most recent retrospective study in the US, to compare HTPMCs with conventional crowns with local anaesthetic (LA) and crown preparation, show a similar success rate for PMCs placed with the traditional technique or the Hall technique.^8^

A recent five-year randomised controlled trial looking at performance of HTPMCs compared to conventional restorations stated that sealing in caries by the Hall technique significantly outperformed GDPs' standard restorations in the long term.^9^ A more recent report^10^ following the teeth to exfoliation, showed that HTPMCs had a better survival rate over lifetime of the teeth than standard restorations, with less major and minor failures.

A recent study in Germany^11^ proved HTPMCs to be more cost effective than conventional restorations as they allowed teeth to be retained for longer.

There appears to be a high acceptability of HTPMCs among children and parents in the UK,^12^ and New Zealand.^13^ Parental perception is that they stay in longer than conventional restorations, and parents are happy with their appearance.^13^ Another recent investigation suggests that the majority of patients, parents and clinicians preferred the Hall technique to conventional restorations.^9^ Perhaps clinicians prefer HTPMCs because it has been found that children's behaviour was better compared to a more traditional approach.^14^ One very recent study^15^ found that children preferred crowns to restorations, and parents preferred tooth coloured restorations. Perhaps the only downside to HTPMCs is their appearance.

Use of HTPMCs by GDPs is increasing. In 2003, Threlfall *et al*. reported that despite national guideline recommendations, GDPs in the UK were not found to be routinely using PMCs,^16^ but by 2011 almost half of Scottish GDPs surveyed were using the Hall technique to fit PMCs in practice.^17^

Authors of a recent update of HTPMC usage^18^ suggest that recommendations be set up to increase conservative management of caries, including the use of the Hall technique.

The Hall technique was introduced to the undergraduate paediatric dentistry curriculum before 2010^19^ and is now taught in all UK and New Zealand dental schools, and in some dental schools across Europe^18^ so we can anticipate that usage by GDPs will increase as more students graduate having learned how to use it.

In a recent study of European postgraduate students in paediatric dentistry, HTPMCs were chosen as an option more often for anxious children than children who were not anxious, ie, not as the treatment of choice for non-anxious children.^20^ Similar results were found in their UK study, a study of postgraduates in paediatric dentistry.^21^ This would suggest they were using HTPMCs for their ease of use, as they are less traumatic for the patient, but still favoured a more traditional approach with local anaesthesia and caries removal as their treatment of choice.

Although Hall technique usage is increasing in the UK and Europe, there remains some controversy around the use of HTPMCs in Canada and the US.^22^,^23^ One of the issues mentioned being that the randomised controlled trial on effectiveness of HTPMCs only compared HTPMCs to GDPs' usual restoration^9^ and that the gold standard of a conventional crown prep with LA and caries removal should still be considered best practice. However, as the recent retrospective study^24^ to compare conventional crowns and HTPMCs found there to be no difference, it would seem preferable to use the technique which causes least stress to the patient, ie, HTPMC.^18^

Further studies are currently taking place to compare cost effectiveness of HTPMCs with conventional restorations and to compare success and cost effectiveness of atraumatic restorative treatment (ART) against HTPMCs.^26^

Several studies have been carried out to prove the effectiveness, cost effectiveness and acceptability of HTPMCs compared to conventional restorations, but there has been little research in terms of what clinical circumstances are appropriate.

This study aimed to look at use of the HTPMCs by specialists in paediatric dentistry in order to create a picture of what specialists consider appropriate cases, which might be useful for dentists who are just starting to use the Hall technique.

For instance the authors wanted to know whether specialists would place HTPMCs on the restorable teeth when a child requires general anaesthesia for extractions for non-restorable teeth, rather than extracting all carious teeth. This would suggest a high level of confidence in the longevity of HTPMCs. It is also interesting to know whether all specialists are using separators, and whether they sometimes fit HTPMCs without taking radiographs.

There are currently 32,900 GDPs in the UK,^27^ and 242 specialists in paediatric dentistry.^28^ A survey of paediatric specialists is therefore looking at a small minority of dentists that could use HTPMCs. As specialists who spend their time treating only children it is useful to gain from their experience. As there are so few specialists in paediatric dentistry compared to GDPs, it is evident that the majority of children will be treated in a primary care setting by a GDP. Welbury describes a reluctance by some older practitioners to use HTPMCs,^19^ and suggests that the use of HTPMCs is to be encouraged in a wider population of dentists treating children, as HTPMCs are easier to tolerate, and quicker to place, and their efficacy has been proved by randomised controlled trials.^18^

Innes describes how specialists in paediatric dentistry are more likely to choose a HTPMC, than a GDP would for the same carious cavity,^18^ and would ask of those who don't use HTPMCs 'why not?' as the evidence clearly show their advantage. To encourage more GDPs to use HTPMCs, it might be useful for them to understand the clinical circumstances when a specialist would use them.

## Materials and methods

This study involved a questionnaire-based online survey (supplementary online only information), using SelectSurvey.NET. Twenty-one questions were asked, giving a multiple choice answer, each including free space for comments. This method was chosen to create a survey that was user friendly, but also allowed comments to give a deeper understanding of some of the situations.

There was a section for demographics, including which part of the country, and which clinical setting the respondents worked in, job title, and how many years they had been practising as a specialist. Questions about use of HTPMCs included whether this technique was the treatment of choice or a treatment option for different types of carious lesions, how long they had been using the technique for, and whether they placed them under inhalation sedation and/or general anaesthesia. They were also asked about use of separators, radiographs and medical contraindications. The final section of the questionnaire was about perceived suitable settings for use of HTPMCs and reasons they are not used, for those who never use them.

A pilot study was carried out on a regional group of paediatric dental specialists and consultants to determine ease of completion. Ethical approval for the study was gained from the University of Liverpool. The online questionnaire was then sent to all paediatric dental specialists in the UK who are members of the British Society of Paediatric Dentistry (97% of all UK paediatric dental specialists) by email in July 2014. This was an anonymous opt-in survey, and therefore no consent was required. A covering letter was sent with the survey, explaining why it was being done, and that it was being sent to all specialists in paediatric dentistry. The survey was sent out again two months later to increase response rate. Descriptive data analysis was carried out using the SelectSurvey.NET. A small number of comments were useful to add a qualitative component to the study.

## Results

Ninety-four of the specialists to whom the survey was sent completed the questionnaire, giving a response rate of 41%.

### Demographics

Ninety percent (85) of respondents worked in the National Health Service (NHS), 1% (1) worked in private practice, and 9% (8) in a mixed setting. Sixty-five percent (61) worked in a dental teaching hospital, and 37% (35) in the community dental service. Fifty-three percent (50) were working as consultants or honorary consultants. All areas of the UK apart from the East Midlands, and East England were represented by the responses.

### Use of HTPMCs

Table 1 shows usage of HTPMCs among respondents in the treatment of primary carious molars. The majority of respondents 58%, (54) considered HTPMCs as a treatment option for carious primary teeth, while 23% (21) considered it the option of choice when it comes to restoring these teeth. Table 2 indicates how long respondents have been using the technique for, with 60% (53) having used the technique for more than five years. Table 3 shows the use of HTPMCs for occlusal and interproximal carious lesions, both cavitated and non-cavitated.

Sixty-seven percent (61) sometimes used orthodontic separators, 15% (14) always used separators, and 10% (9) rarely used them. Only 7% (6) never used them.

Ninety-eight percent (92) felt that HTPMCs were suitable for use in specialist paediatric settings, both hospital and community, 97% (91) felt they were suitable for both general dental practice and postgraduate training, and 90% (85) felt they were suitable for undergraduate teaching. Table 4 shows how HTPMCs were taught by specialists to students, therapists, trainees and other groups.

### Treatment planning

Seventy-six percent (68) would plan for fitting HTPMCs under inhalation sedation, and 26% (23) would plan to fit them under general anaesthesia. However, several respondents would prefer to remove caries first if fitting under general anaesthesia. Reasons given for not using this technique under general anaesthesia included avoiding interference with the freeway space.

Forty-six percent (42) would fit a HTPMC before general anaesthesia for extraction of other teeth. Several said that any teeth with a history of caries are extracted under general anaesthesia, and another that any teeth that have HTPMCs on them would be extracted under general anaesthesia. Several mentioned that the decision to fit HTPMCs before (or during) general anaesthesia would depend on the extent of caries and the ability to take bitewing radiographs.

Fifty-nine percent (54) responded that they would always take a radiograph.

Thirty-four percent (30) said they believed there were no medical contraindications to the use of HTPMCs. Of those who believed there are medical contraindications, the majority cited immunocompromised and cardiac conditions. Other conditions mentioned were leukaemia, brain tumours, and severe special needs.

Only 4% (4) of respondents indicated that they never use HTPMCs.

The final question of the survey aimed to uncover the reasons why some specialists do not use HTPMCs. Some respondents gave more than one reason for not using the technique. Three respondents felt that there is not sufficient evidence to warrant their use, two respondents didn't use them because they had not been taught the technique, and one person said they didn't feel confident with the technique. One respondent replied that, in their opinion, most patients don't want them, and several prefer to ensure all caries is removed.

## Discussion

Although a response rate of 41% is seemingly low, it compares favourably to a recent study carried out by Cunningham *et al*.^29^ which looked at response rates to an online survey by physician specialists in Canada. It is claimed that 35% is a comparable response rate for online surveys for physician specialist-based surveys. This does not detract from the issue of response bias. The respondents were spread across the whole of the UK, and worked in a mixture of settings (predominantly teaching hospitals and community clinics which is where most specialists work) and 90% worked for the NHS, which would suggest the respondents were a representative sample.

The results suggest that HTPMCS are widely used among specialists in paediatric dentistry. However, HTPMCs are still not used by the majority as the treatment of choice for symptomless carious molars. Most respondents viewed them as a treatment option, with almost a quarter who only used them when they were unable to provide a conventional restoration. It is not clear why more specialists do not use HTPMCs as the treatment of choice, given their success rate and ease of fitting compared to conventional restorations requiring local anaesthesia and caries removal. However, specialists do seem to approve of their use in general, with 90% saying that they believed they should be taught to undergraduate dental students. As HTPMCs become used more often, it will be interesting to see if they become the treatment of choice for carious primary molars.

It would appear that HTPMCs are being used more often in cavitated lesions than non-cavitated and more often in interproximal than occlusal lesions. This would appear to be in accordance with the Hall technique manual^1^ which advises their use for two surface lesions or extensive one surface lesions. It is interesting to note that a significant number of respondents use HTPMCs sometimes for non-cavitated and cavitated occlusal cavities, in contrast with the manual^1^ which suggests sealant only for non-cavitated, and partial caries removal and sealant for cavitated occlusal cavities. Perhaps this is due to the ease of fitting HTPMCs compared to partial caries removal.

There appears to be a high level of confidence in the effectiveness of HTPMCs, as demonstrated by the fitting of HTPMCs before general anaesthesia for extraction of other teeth. One individual even suggested that they might fit HTPMCs onto non-carious teeth in a high risk patient during general anaesthesia. This would suggest that some specialist clinicians are using the Hall technique as a purely preventive measure. Interestingly, several specialists would extract symptomless teeth with a HTPMC on them if extracting under GA, demonstrating less faith in the longevity of HTPMCs.

In the manual^1^ it is considered best practice to take a radiograph before fitting a HTPMC, but this is not always possible with very young children. Specialists appear to be prepared to fit HTPMCs without radiographs when convinced it is appropriate. From the comments section it was apparent that several respondents would feel comfortable fitting a HTPMC on a child without taking radiographs, if the child was unable to tolerate bitewings, providing the tooth was symptomless, and clinically they were able to rule out pulpal involvement.

It emerged that, although very few in number, there are still some specialists who are reluctant to use HTPMCs. The main reason for non-use was given as lack of training or lack of confidence, along with a lack of confidence in the available evidence.

## Conclusion

The use of HTPMCs is gaining momentum, not only in general practice, but also in their use by specialists in paediatric dentistry in the UK. There is a difference in the way that specialists use HTPMCS, with some using them as the treatment of choice for all cavitated carious primary molars, and a very small number who never use them at all. Most of the specialists view them as a treatment option rather than the treatment of choice, which is surprising, given their effectiveness and ease of use. Some specialists are using HTPMCs as a preventive approach, fitting them on primary molars with non-cavitated caries, and even to caries free teeth under GA. There is a high level of confidence in HTPMCs among specialists, as demonstrated by them being fitted before, and during general anaesthesia.

The results suggest that specialists are pragmatic in their approach to use of HTPMCs. Some of them were happy to fit HTPMCs without radiographs when they were unable to obtain them. Most use separators, but not all the time; their use is not a prerequisite for fitting HTPMCs.

The majority of specialists believe that the Hall technique should be taught to undergraduate students, and used in a wide variety of settings, suggesting they consider HTPMCs to be a suitable treatment option for carious primary molars, for use by GDPs. As the majority of specialists advocate teaching the use of HTPMCs to students and trainees, and they are now being taught in all UK dental schools,^18^ the number of dentists trained in the technique will continue to increase year on year.

## Figures and Tables

**Table 1:** When do you use/plan for Hall technique preformed crowns? (N = 93)

When HTPMCs are used	Percentage (number) of respondents
Treatment option for carious primary molar	58% (54)
Treatment of choice for carious primary molar	23% (21)
Only when unable to use a conventional restoration in a carious primary molar	15% (14)
Never	4% (4)

**Table 2:** How long have you been using Hall technique preformed metal crowns? (N = 89)

Length of time using HTPMCs	Percentage (number) of respondents
Over 5 years	60% (53)
4 years	18% (16)
3 years	15% (13)
2 years	7% (6)
1 year or less	1% (1)

**Table 3:** Use of Hall technique preformed crowns

	Would you plan to use HTPMC for a non-cavitated occlusal carious lesion? (N = 90)	Would you plan to use HTPMC for a cavitated occlusal carious lesion? (N = 91)	Would you plan to use HTPMC for an apparently non-cavitated interproximal carious lesion? (N = 91)	Would you plan to use HTPMC for a cavitated interproximal carious lesion? (N = 90)
Always	0%	2% (2)	8% (7)	21% (19)
Sometimes	43% (39)	70% (64)	60% (55)	67% (60)
Rarely	30% (27)	19% (17)	19% (17)	9% (8)
Never	26% (23)	8% (7)	12% (11)	2% (2)
N/A	1% (1)	1% (1)	1% (1)	1% (1)

**Table 4:** Do you teach use of Hall technique preformed metal crowns to any of the following? (N = 94)

	Percentage (number) of respondents
Undergraduate dental students	49% (46)
Dental therapists	41% (39)
Hygiene therapists	14% (13)
Newly qualified dentists	63% (59)
Specialist trainees	64% (60)
Post CSST (in consultant training)	43% (40)
Postgraduate students	40% (38)
